# Socioeconomic differences in caesarean section – are they explained by medical need? An analysis of patient record data of a large Kenyan hospital

**DOI:** 10.1186/s12939-020-01215-2

**Published:** 2020-07-08

**Authors:** Lisa van der Spek, Sterre Sanglier, Hillary M. Mabeya, Thomas van den Akker, Paul L. J. M. Mertens, Tanja A. J. Houweling

**Affiliations:** 1 Department of Public Health, Erasmus MC, University Medical Center Rotterdam, Rotterdam, The Netherlands; 2 Department of Reproductive Health, Moi University School of Medicine and Gynocare Womens and Fistula Hospital, Eldoret, Kenya; 3 Department of Obstetrics and Gynaecology, Leiden University Medical Center, Leiden, The Netherlands; 4 Athena Institute, Vrije Universiteit Amsterdam, Amsterdam, The Netherlands; 5 School of Public Health, Moi University, Eldoret, Kenya

**Keywords:** Delivery, Caesarean section, Maternity services, Developing countries, Obstetrics and gynaecology, Epidemiology, General obstetric, Pregnancy, Health equity, Socioeconomic factors, Africa, Clinical category, General obstetrics

## Abstract

**Background:**

Caesarean section (C-section) rates are often low among the poor and very high among the better-off in low- and middle-income countries. We examined to what extent these differences are explained by medical need in an African context.

**Methods:**

We analyzed electronic records of 12,209 women who gave birth in a teaching hospital in Kenya in 2014. C-section rates were calculated by socioeconomic position (SEP), using maternal occupation (professional, small business, housewife, student) as indicator. We assessed if women had documented clinical indications according to hospital guidelines and if socioeconomic differences in C-section rates were explained by indication.

**Results:**

Indication for C-section according to hospital guidelines was more prevalent among professionals than housewives (16% vs. 9% of all births). The C-section rate was also higher among professionals than housewives (21.1% vs. 15.8% [OR 1.43; 95%CI 1.23–1.65]). This C-section rate difference was largely explained by indication (4.7 of the 5.3 percentage point difference between professionals and housewives concerned indicated C-sections, often with previous C-section as indication). Repeat C-sections were near-universal (99%). 43% of primary C-sections had no documented indication. Over-use was somewhat higher among professionals than housewives (C-section rate among women without indication: 6.6 and 5.5% respectively), which partly explained socioeconomic differences in primary C-section rate.

**Conclusions:**

Socioeconomic differences in C-section rates can be largely explained by unnecessary primary C-sections and higher supposed need due to previous C-section. Prevention of unnecessary primary C-sections and promoting safe trial of labor should be priorities in addressing C-section over-use and reducing inequalities.

**Tweetable abstract:**

Unnecessary primary C-sections and ubiquitous repeat C-sections drive overall C-section rates and C-section inequalities.

## Background

Caesarean section (C-section) rates are rapidly rising in low and middle income countries [1] and can reach very high levels among women of higher socioeconomic position (SEP) [2]. At the same time, unmet need for C-section among poor women in these countries is usually high. While C-section rates remain low in most Sub-Saharan African countries, they are gradually increasing, and socioeconomic differences in C-section rates are substantial [3]. In Kenya, for example, the C-section rate ranges from 2.4% in the poorest quintile to 19% in the richest quintile, as estimated from a nationally representative survey conducted in 2014 [4].

While the surgery can be life-saving when medically indicated, C-section rates above 10% at the population level are not associated with improved maternal and newborn outcomes [5, 6]. On the contrary, unnecessary C-sections are associated with higher risks of adverse outcomes for woman and baby compared with vaginal birth [7, 8].

While socioeconomic inequalities in C-section rates are well-documented, it remains unknown to what extent they can be explained by higher medical need among better-off women. Medical need for C-section might arguably differ between socioeconomic groups, for example due to differences in age and parity. Individual-level data on clinical indication for C-section are often not available or accessible in low- and middle-income countries [9, 10], let alone in combination with information on socioeconomic position.

Our study aimed to address this paucity of evidence by describing and explaining socioeconomic inequalities in C-section using clinical record data of an academic referral hospital in Kenya. Specifically, we aimed to examine clinical indications for C-section and under- and over-use of C-section across socioeconomic groups, and the role of medical need for C-section in explaining socioeconomic inequalities in C-section rates.

## Methods

### Study setting

Our study was conducted in the public wing of a large academic referral hospital in Kenya. The maternity department consisted of an antepartum ward (29 beds), labor ward (18 beds), postpartum ward (35 beds), neonatology unit (max. 160 beds), and a hostel (45 beds, 24-h observation of post-partum women without complications). Maternity care in the hospital, as in the rest of Kenya, was officially free as of June 1st, 2013.

### Study population and data collection

All women who gave birth in the public wing of the hospital between 1 January and 31 December 2014 were included in our study. Excluded were births of fetuses with an estimated weight below 650 g (21 deliveries) because C-sections were not performed in this hospital in these women. All women who gave birth at the hospital, were registered in an electronic Delivery Database after discharge. The Delivery Database contained a digitalized version of parts of the manual patient file that all women received, and included the following variables: patient number, admission date and time, discharge date, maternal age, parity, maternal occupation, ICD codes, multiple gestation, mode of birth, outcome mother, outcome infant, birth weight, name of ANC clinic the mother attended, referring facility, and reason for referral. Medical record officers digitalized the manual files and coded data according to the International Classification of Diseases and Related Health Problems 10th edition (ICD-10) and the International Classification of Procedures in Medicine [11, 12]. C-sections were also registered in a Surgery Database: a digitalized version of the Surgery Book, which contained all surgeries at the maternity department. We obtained anonymised versions of the Delivery Database and Surgery Database for our analyses. We linked the Delivery Database and Surgery Database on the basis of patient number, in combination with other variables in the databases where needed. To verify the accuracy of the electronic databases, we conducted a detailed review of a random selection of manual files (case notes). Hundred fifty women who gave birth (either vaginally or by CS) and 50 women who gave birth specifically by CS in MTRH in 2014 were randomly selected from the Delivery Database. For 131 (82 vaginal, 49 CS) out of the 200 women, we were able to retrieve the manual file. A detailed review of the manual files was done by LvdS and SS to assess whether or not women had a documented CS indication, using procedures described below.

### Definition of study outcome and determinants

The study outcome was defined mode of birth (C-section vs. vaginal birth). Records were included as C-section if the Delivery Database noted C-section as mode of birth and/or included an ICD code for C-section, and/or if the woman was registered in the Surgery Database as having delivered via C-section. All other records were included as vaginal births.

Socioeconomic position was defined on the basis of occupation of the woman giving birth. Occupation was registered in nine categories, which we summarized into four categories as follows: housewives (housewife, unemployed), small business (small business, casual laborer, farmer), professional (professional, government employee, private employee), and student (student/pupil). Maternal age was registered in years and categorised as follows: < 16, 16–20, 21–25, 26–30, 31–35, 36–40, and > 40 years.

The Robson classification has been developed to compare C-section rates across hospitals, and provides a starting point for accessing hospital-based C-section rates. To categorize women according to the Robson classification [13], we used information on parity, gestational age (in weeks), presentation (cephalic/ breech/ other non-cephalic presentation), number of fetuses, and previous C-section (yes/no) from the Delivery Database. As we had no information on spontaneous vs. induced labor, we used an adapted version of the Robson classification (see Table 6). 2845 women (23%) could not be classified into a Robson category because of missing information on gestational age. We developed two additional groups (Group 11: All nulliparous women, singleton cephalic, gestational age unknown; Group 12: All multipara, singleton cephalic, gestational age unknown) to address this problem.

Finally, parity was not always consistently recorded – sometimes as parity before birth, sometimes as parity after birth. As the proportion of women with parity recorded as zero led to an implausibly low estimate of the proportion of nulliparous women (5.5%), we included all women with parity recorded as zero or one as nulliparous, which might have led to an over-estimation of nulliparous women and a dilution of the effect of parity on mode of birth.

For each woman, we determined whether she had an indication for C-section according to the clinical guidelines of the hospital [14]. We obtained these guidelines from the maternity department and translated these into ICD codes and other information necessary to determine C-section indication. Table S1 provides a full overview of the hospital guidelines, information necessary and information available to determine clinical indication post-hoc. We used the ICD codes and other information in the Delivery and Surgery Databases to determine if a woman had a C-section indication according to the guidelines. The information in the databases was not always detailed enough to conclusively determine if a woman had a clinical indication. For example, fetal anomaly incompatible with spontaneous vertex birth (SVB) is a C-section indication according to the hospital guidelines. An ICD code for foetal abnormality exists, but this does not clarify whether the anomaly was incompatible with SVD. As another example, previous C-section is an indication for C-section according to the hospital guidelines in the case of two or more previous C-sections. The ICD codes contained whether a woman had previous C-section, but did not provide details on the number of previous C-sections. In such cases, where part of the information was missing, we used the precautionary principle and assumed that the woman had a C-section indication. For comparative purposes, we also determined for each woman if she had a C-section indication when using the Kenyan national guidelines [15, 16], the Dutch [17–24] and English [25–29] guidelines.

### Analyses

First, we calculated the C-section rate for the total population and by socioeconomic position and other background characteristics. Then, we calculated the percentage of women with C-section indication and examined the determinants of C-section indication using logistic regression analysis. Next, we calculated the C-section rate among women with and without C-section indication and the percentage of C-section deliveries without clinical indication. Then, we examined determinants of C-section using logistic regression analysis. Using multivariable logistic regression analyses, we examined if socioeconomic inequalities in the odds of C-section were explained by differences in medical need for a C-section (defined as C-section indication according to the hospital guidelines), previous C-section, maternal age and parity. We also divided the population into the Robson groups, and examined if there were socioeconomic inequalities in C-section rate within Robson groups. We analyzed the data using Stata 13 (Stata, College Station, TX, USA).

## Results

In 2014, 12,209 women gave birth in the hospital (Table 1). Most women (58%) were housewives; a minority (11%) had a professional occupation. Professional women tended to be older than women of other socioeconomic groups. Nearly 50% of women had a parity of 0 or 1, while parity above four was rare, especially among students and professionals. Previous C-sections were more common among professionals than in other socioeconomic groups. Only a tiny fraction (1%) of women were referral patients.

**Table 1:** Distribution of the study population by background characteristics, and C-section rate by background characteristics

	Distribution of the study population by background characteristics							C-section rates by background characteristics	
	Total (*n* = 12,209)		Housewife	Small business	Professional	Student	Missing		
	n	%	%	%	%	%	%	n	%
**Characteristics of the mother**									
All deliveries ^a^	12,209	100						2020/12209	16.5
Occupation									
Housewife	7129	58						1125/7129	15.8
Small business	2161	18						398/2161	18.4
Professional	1304	11						275/1304	21.1
Student	1375	11						190/1375	13.8
Missing	240	2						32/240	13.3
Age of mother									
< 16 years	61	1	0	0	0	3	1	8/61	13.1
16–20 years	2081	17	17	9	4	41	19	273/2081	13.1
21–25 years	4437	36	38	32	27	46	38	623/4437	14
26–30 years	3373	28	28	33	40	8	27	617/3373	18.3
31–35 years	1452	12	11	16	18	2	12	318/1452	21.9
36–40 years	656	5	5	8	10	0	3	145/656	22.1
> 40 years	124	1	1	2	1	0	0	34/124	27.4
Missing	25	0	0	0	0	0	1	2/25	8
Parity ^b^									
0–1	5915	48	44	39	47	89	60	926/5915	15.7
2–3	5564	46	49	54	49	11	37	976/5564	17.5
> 4	692	6	7	7	4	0	3	114/692	16.5
Missing	38	0	0	0	0	0	1	4/38	10.5
Number of fetuses									
Singleton	11,726	96	96	96	96	96	95	1884/11726	16.1
Multiple gestation	228	2	2	2	2	1	2	103/228	45.2
Missing	255	2	2	2	2	2	3		
Previous C-section									
No	11,605	95	95	94	92	99	96	1419/11605	12.2
Yes	604	5	5	6	8	1	4	601/604	99.5
Referral patient									
No	12,000	98	98	98	99	99	99	1947/12000	16.2
Yes	209	2	2	2	1	1	1	73/209	34.9
Antenatal Care Attended									
No	269	2	2	2	3	2	7	40/269	14.9
Yes	11,940	98	98	98	98	98	93	1980/11940	16.6
**Characteristics of the infant**									
Position fetus									
Cephalic	11,883	97.3	98	97	97	98	98	1743/11883	14.7
Breech	253	2.1	2	3	3	2	2	211/253	83.4
Other	73	0.6	1	1	1	1	0	66/73	90.4
Gestational age (in weeks)									
Very preterm (28–31)	241	2	2	2	2	3	2	43/241	17.8
Moderate to late preterm (32–36)	1073	8.8	9	9	6	9	6	196/1073	18.3
Term (> 36)	7725	63.3	62	64	70	63	66	1302/7725	16.9
Missing	3170	26	27	26	22	25	25	479/3170	15.1
Birthweight singletons (11,726 infants)									
Very low (650–1499)	196	1.7	2	2	1	2	3	29/196	14.8
Low (1500–2499)	1070	9.1	9	9	7	12	9	206/1070	19.3
Normal (2500+)	10,148	86.5	87	86	90	84	80	1592/10148	15.7
Missing	312	2.7	2	3	2	3	8	57/312	18.3
Birthweight multiple gestation (419 infants in 228 births)									
Very low (650–1499)	46	11.0	11	12	21	3	13	8/46	17.4
Low (1500–2499)	212	50.6	47	59	47	72	13	103/212	48.6
Normal (2500+)	161	38.4	42	29	32	24	88	69/161	42.9

^a^Fetuses with a birthweight < 650 g were excluded

^b^Parity was not always consistently recorded - sometimes as parity before delivery, sometimes as parity after delivery. Therefore, we combined into one category women with parity recorded as zero and women with parity recorded as one

The C-section rate was 16.5%, varying from 21.1% among professionals to 15.8% among housewives, and 13.8% among students. The rate increased with maternal age, from 13% in the ≤20 years groups to over 27% in the > 40 years group. Among women with a previous C-section, C-section was nearly universal (99%).

The prevalence of clinical indication for C-section was highest among professionals (16% of all births among professionals) and lowest among students (9%), with housewives being in-between (11%) (Table 2). The higher odds of indication in professionals compared with housewives (OR 1.48; 95%CI 1.25–1.75) was largely explained by maternal age, parity, and previous C-section (aOR 1.17; 95%CI 0.93–1.48), and only by previous C-section when professionals were compared with students (Table S2).

**Table 2:** Medical indication for C-section according to the hospital guidelines: percentage and determinants

	Women with medical indication		Univariate		Adjusted for maternal age ^a^		Adjusted for parity ^b^		Adjusted for previous C-section		Adjusted for all ^a b^	
	n	%	OR [95% CI]	*P* value	OR [95% CI]	*P* Value	OR [95% CI]	*P* Value	OR [95% CI]	*P* Value	OR [95% CI]	*P* Value
Total population	1433/12209	11.7	–	–	–	–	–	–	–	–	–	–
*Occupation*												
Housewife	797/7129	11.2	1		1		1		1		1	
Small business	295/2161	13.7	1.26 (1.09;1.45)	0.0018	1.16 (1.00;1.34)	0.0454	1.24 (1.08;1.44)	0.0028	1.21 (1.00;1.46)	0.0499	1.17 (0.97;1.41)	0.1071
Professional	205/1304	15.7	1.48 (1.25;1.75)	0.0000	1.31 (1.11;1.55)	0.0016	1.5 (1.27;1.77)	0.0000	1.33 (1.07;1.66)	0.0117	1.17 (0.93;1.48)	0.1672
Student	117/1375	8.5	0.74 (0.60;0.91)	0.0035	0.93 (0.76;1.15)	0.5087	0.81 (0.65;0.99)	0.0411	1.19 (0.95;1.48)	0.1294	1.19 (0.94;1.49)	0.1402
Overall *p*-value				0.0000		0.0044		0.0000		0.0281		0.1832
*Maternal age*												
< 16 years	4/61	6.6	1				1		1		1	
16–20 years	171/2081	8.2	1.28 (0.46;3.56)	0.6417	–	–	1.27 (0.45;3.54)	0.6509	1.09 (0.39;3.04)	0.8705	1.12 (0.40;3.15)	0.8330
21–25 years	413/4437	9.3	1.46 (0.53;4.05)	0.4646	–	–	1.46 (0.53;4.05)	0.4656	1.02 (0.37;2.83)	0.9698	1.1 (0.39;3.09)	0.8534
26–30 years	461/3373	13.7	2.26 (0.81;6.25)	0.1175	–	–	2.33 (0.84;6.48)	0.1035	1.13 (0.40;3.13)	0.8207	1.37 (0.48;3.87)	0.5541
31–35 years	239/1452	16.5	2.81 (1.01;7.81)	0.0480	–	–	2.99 (1.07;8.35)	0.0370	1.17 (0.42;3.30)	0.7601	1.63 (0.57;4.69)	0.3655
36–40 years	119/656	18.1	3.16 (1.12;8.87)	0.0291	–	–	3.51 (1.24;9.95)	0.0184	1.19 (0.41;3.44)	0.7425	1.97 (0.66;5.84)	0.2228
> 40 years	25/124	20.2	3.6 (1.19;10.86)	0.0231	–	–	4.17 (1.36;12.80)	0.0126	2.16 (0.68;6.82)	0.1896	4.24 (1.29;13.90)	0.0173
Overall *p*-value				0.0000				0.0000		0.2171		0.0001
*Parity*												
0–1	557/5915	9.4	1		1				1		1	
2–4	795/5564	14.3	1.6 (1.43;1.80)	0.0000	1.21 (1.06;1.38)	0.0037	–	–	0.62 (0.54;0.73)	0.0000	0.5 (0.42;0.60)	0.0000
> 4	78/692	11.3	1.22 (0.95;1.57)	0.1178	0.64 (0.48;0.85)	0.0020	–	–	0.8 (0.58;1.09)	0.1581	0.48 (0.34;0.70)	0.0000
Overall *p*-value				0.0000		0.0000				0.0000		0.0000
*Previous C-section*												
No	829/11605	7.1	–	–	–	–	–	–	–	–	–	–
Yes	604/604	100	–	–	–	–	–	–	–	–	–	–

^a^Adjustment for age in years; ^b^Adjustment for parity in actual number of births (not in parity categories)

Nearly all women with a C-section indication gave birth accordingly, irrespective of socioeconomic position (Table 3). There were small differences in unmet need: 2.4% of housewives with a C-section indication had a vaginal birth, compared with 1% among professionals. Over-use according to hospital guidelines was somewhat higher among professionals than among housewives: among births without C-section indication, 6.6% (professionals) and 5.5% (housewives) respectively ended up with a C-section.

**Table 3:** C-section and vaginal delivery rate among women with and without clinical indication

	C-section rate among women with indication		Vaginal delivery rate among women with indication		C-section rate among women without indication	
	n	%	n	%	n	%
Total population	1404 /1433	98.0	29 /1433	2.0	616 /10776	5.7
Housewife	778 /797	97.6	19 /797	2.4	347 /6332	5.5
Small business	291 /295	98.6	4 /295	1.4	107 /1866	5.7
Professional	203 /205	99.0	2 /205	1.0	72 /1099	6.6
Student	115 /117	98.3	2 /117	1.7	75 /1258	6.0

For around 30% of C-sections there was no C-section indication (Table 4); this was similar (27% [13/49]) in our review of manual patient files. Previous C-section as indication accounted for 30% of C-sections (22% when considering previous C-section as only indication, 30% when also including multiple indications that included previous C-section). This proportion was higher among professionals (37% when including multiple indications) than among housewives (31%).

**Table 4:** Distribution of C-section deliveries according to indication

	% with no C-section indication		% with only previous C-section as indication		% with multiple indications including PCS		% with only foetal distress as indication		% with only prolonged labour as indication		% with other indication		% with multiple indications excluding PCS		Total	
	n	%	n	%	n	%	n	%	n	%	n	%	n	%	n	%
Total population	616/2020	30.5	451/2020	22.3	150/2020	7.4	245/2020	12.1	332/2020	16.4	145/2020	7.2	81/2020	4	2020	100
Housewife	347/1125	30.8	265/1125	23.6	81/1125	7.2	127/1125	11.3	174/1125	15.5	86/1125	7.6	45/1125	4	1125	100
Small business	107/398	26.9	94/398	23.6	40/398	10.1	52/398	13.1	62/398	15.6	29/398	7.3	14/398	3.5	398	100
Professional	72/275	26.2	78/275	28.4	23/275	8.4	36/275	13.1	39/275	14.2	19/275	6.9	8/275	2.9	275	100
Student	75/190	39.5	7/190	3.7	4/190	2.1	28/190	14.7	54/190	28.4	11/190	5.8	11/190	5.8	190	100

*PCS* previous C-section

**Fig. 1:**
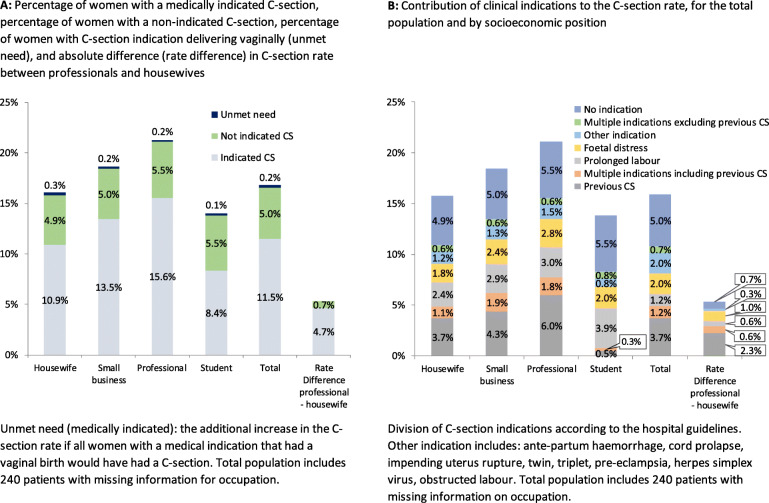
C-section rate by clinical indication and unmet need, for total population and by socioeconomic position

The higher C-section rate among professionals compared with other socioeconomic groups was mostly due to higher medical need, while over-use based on hospital guidelines only contributed a little: the C-section rate among professionals (21.5%) was built up of 15.6% indicated C-sections plus 5.5% not indicated C-sections (of all births), compared with 10.9% indicated plus 4.9% non-indicated C-sections among housewives (Fig. 1a). In other words, the C-section rate difference between professionals and housewives of 5.3 percentage points (pp) was for 4.7 pp. due to indicated C-sections. These patterns were similar when using the Kenyan, Dutch and English guidelines (Table S3). The C-section rate difference of 4.7 pp. due to indicated C-sections consisted for 2.9 pp. of indication related to previous C-sections (Fig. 1b).

The odds of a C-section were 1.43 (95%CI 1.23–1.65) times higher among professionals compared with housewives (1.67 [95%CI 1.36–2.04] times higher compared with students) (Table 5 and Table S4). The higher C-section rate among professionals compared with housewives was not explained by multiple births, presentation, or gestational age. It was substantially explained by C-section indication, previous C-section, maternal age, and parity. The combination of the above variables nearly fully explained the higher C-section rate among professionals compared with housewives (aOR 1.08; 95%CI 0.83–1.40). Previous C-sections explained the higher C-section rate among professionals compared with students.

**Table 5:** Determinants of C-section: univariate and multivariable analysis

	Univariate		Adjusted for clinical indication		Adjusted for previous C-section		Adjusted for multiple birth, presentation, and gestational age		Adjusted for maternal age and parity ^a^		Adjusted for maternal clinical indication, previous C-section, age, and parity ^a^		Adjusted for all ^a^	
	OR [95% CI]	*P* value	OR [95% CI]	*P* Value	OR [95% CI]	*P* value	OR [95% CI]	Pvalue	OR [95% CI]	*P* Value	OR [95% CI]	*P* Value	OR [95% CI]	*P* value
*Occupation*														
Housewives	1		1		1		1		1		1		1	
Small business	1.2 (1.06;1.37)	0.0038	1.07 (0.87;1.33)	0.5140	1.15 (0.99;1.34)	0.0644	1.12 (0.96;1.31)	0.1412	1.12 (0.98;1.27)	0.0894	1.03 (0.83;1.29)	0.7762	1.04 (0.79;1.37)	0.7823
Professional	1.43 (1.23;1.65)	0.0000	1.24 (0.96;1.61)	0.0925	1.31 (1.09;1.56)	0.0032	1.41 (1.18;1.68)	0.0001	1.22 (1.04;1.42)	0.0117	1.08 (0.83;1.41)	0.5451	1.11 (0.80;1.54)	0.5431
Student	0.86 (0.72;1.01)	0.0656	1.11 (0.86;1.42)	0.4373	1.16 (0.98;1.38)	0.0873	0.87 (0.72;1.07)	0.1850	0.98 (0.82;1.16)	0.8004	1.07 (0.82;1.38)	0.6282	1.02 (0.73;1.43)	0.8920
Overall *p*-value		0.0000		0.3726		0.0105		0.0002		0.0413		0.9102		0.9420
*Age of Mother*														
< 16 years	1		1		1		1		1		1		1	
16–20 years	1 (0.47;2.13)	0.9993	0.76 (0.28;2.11)	0.6015	0.91 (0.43;1.93)	0.8050	1.03 (0.36;2.95)	0.9617	1 (0.47;2.12)	0.9943	0.75 (0.27;2.08)	0.5789	1.24 (0.20;7.85)	0.8210
21–25 years	1.08 (0.51;2.29)	0.8361	0.75 (0.27;2.06)	0.5748	0.87 (0.41;1.83)	0.7075	1.11 (0.39;3.17)	0.8438	1.11 (0.53;2.36)	0.7778	0.78 (0.28;2.16)	0.6326	1.24 (0.20;7.83)	0.8183
26–30 years	1.48 (0.70;3.14)	0.3020	0.78 (0.29;2.16)	0.6385	0.93 (0.44;1.97)	0.8522	1.51 (0.53;4.32)	0.4379	1.65 (0.78;3.49)	0.1936	0.91 (0.32;2.54)	0.8536	1.53 (0.24;9.73)	0.6545
31–35 years	1.86 (0.87;3.95)	0.1073	0.96 (0.34;2.66)	0.9331	1.05 (0.49;2.24)	0.8999	1.82 (0.64;5.23)	0.2645	2.23 (1.04;4.76)	0.0389	1.38 (0.48;3.92)	0.5499	2.26 (0.35;14.63)	0.3918
36–40 years	1.88 (0.87;4.04)	0.1063	0.73 (0.25;2.12)	0.5670	0.93 (0.42;2.03)	0.8476	1.82 (0.62;5.29)	0.2738	2.48 (1.14;5.38)	0.0221	1.21 (0.40;3.62)	0.7387	1.18 (0.17;8.22)	0.8670
> 40 years	2.5 (1.08;5.81)	0.0326	1.35 (0.41;4.49)	0.6238	1.77 (0.74;4.22)	0.1981	2.09 (0.65;6.69)	0.2149	3.68 (1.56;8.69)	0.0030	2.96 (0.86;10.21)	0.0864	2.55 (0.31;21.12)	0.3847
Overall *p-*value		0.0000		0.3577		0.0518		0.0000		0.0000		0.0003		0.0314
*Parity*														
0–1	1		1		1		1		1		1		1	
2–4	1.15 (1.04;1.26)	0.0066	0.58 (0.49;0.69)	0.0000	0.58 (0.51;0.65)	0.0000	1.19 (1.06;1.34)	0.0039	0.88 (0.79;0.98)	0.0224	0.46 (0.37;0.56)	0.0000	0.47 (0.37;0.61)	0.0000
> 4	1.06 (0.86;1.31)	0.5758	0.85 (0.61;1.20)	0.3676	0.81 (0.64;1.04)	0.0932	0.99 (0.75;1.30)	0.9371	0.57 (0.45;0.73)	0.0000	0.54 (0.36;0.81)	0.0027	0.66 (0.39;1.11)	0.1179
Overall *p*-value		0.0250		0.0000		0.0000		0.0121		0.0000		0.0000		0.0000
*Number of babies*														
Singleton	1		1		1		1		1		1		1	
Multiple	4.3 (3.30;5.61)	0.0000	1.88 (1.10;3.22)	0.0220	5.29 (4.01;6.99)	0.0000	1.87 (1.25;2.79)	0.0024	4.11 (3.14;5.38)	0.0000	1.53 (0.85;2.76)	0.1542	0.81 (0.29;2.24)	0.6791
*Presentation foetus*														
Cephalic	1		1		1		1		1		1		1	
Breech	29.23 (20.91;40.86)	0.0000	52.22 (36.04;75.67)	0.0000	36.67 (26.07;51.58)	0.0000	31.17 (20.38;47.66)	0.0000	28.46 (20.33;39.85)	0.0000	53.03 (36.28;77.52)	0.0000	64.44 (40.36;102.88)	0.0000
Other	54.85 (25.13;119.75)	0.0000	131.97 (59.06;294.90)	0.0000	66 (29.97;145.37)	0.0000	55.6 (22.10;139.87)	0.0000	55.46 (25.35;121.34)	0.0000	136.67 (60.72;307.62)	0.0000	131.75 (50.26;345.35)	0.0000
Overall *p*-value		0.0000		0.0000		0.0000		0.0000		0.0000		0.0000		0.0000
*Gestational age (in weeks)*														
Term	1		1		1		1		1		1		1	
Moderate to late preterm (32–36)	1.03 (0.71;1.48)	0.8774	0.76 (0.44;1.32)	0.3301	0.88 (0.59;1.30)	0.5227	0.84 (0.57;1.25)	0.4009	1.12 (0.77;1.62)	0.5535	0.74 (0.42;1.29)	0.2867	0.49 (0.27;0.91)	0.0229
Term (> 36)	0.93 (0.67;1.30)	0.6869	0.7 (0.43;1.15)	0.1601	0.74 (0.52;1.06)	0.0990	0.94 (0.65;1.35)	0.7313	0.99 (0.71;1.39)	0.9635	0.7 (0.42;1.15)	0.1575	0.57 (0.34;0.98)	0.0413
Overall *p*-value		0.4872		0.3329		0.0629		0.4896		0.3736		0.3537		0.0728
*Previous C-section*														
No	1		1				1		1		1		1	
Yes	1438.02 (461.86;4477.31)	0.0000	6.49 (1.95;21.53)	0.0023	–	–	1215.45 (389.63;3791.59)	0.0000	1496.97 (480.29;4665.79)	0.0000	9.83 (2.32;41.72)	0.0019	7.18 (1.64;31.51)	0.0090
*Indication according to guideline MTRH*														
No	1		1		1		1		1		1		1	
Yes	798.51 (547.94;1163.66)	0.0000	–	–	509.4 (341.82;759.12)	0.0000	1060.18 (664.91;1690.44)	0.0000	816.76 (559.06;1193.26)	0.0000	971.52 (602.46;1566.67)	0.0000	680.69 (408.70;1133.70)	0.0000

^a^Multivariable analysis: adjustment for age in years and for parity in actual number of births (not in parity categories)

The combined Robson groups 1 + 2 and Robson group 5 contributed most to the overall C-section rate and to the difference in C-section rate between professionals and housewives (Table 6). The higher C-section rate among professionals (17%) compared with other women (13% among housewives) in Robson groups 1 + 2 (nulliparous women with a full-term pregnancy of a singleton in cephalic presentation) is noteworthy. Differences –albeit smaller- were also observed for Groups 3 + 4 (multiparous women without previous C-section with a term singleton in cephalic presentation) (9.6% vs 7.2%). For other Robson groups the number of women in each SEP group was very small.

**Table 6:** Distribution of the study population according to the Robson Classification and C-section rates per Robson Group, for the total population and by SEP

	Total				Housewives				Small business				Professional				Student			
Group^a^	n in Robson Group	% of population in Robson Group %	C-section rate in Robson Group (%)	Contribution to CS rate of 16.5% (pp)^b^	n in Robson Group	% of housewives in Robson Group	C-section rate in Robson Group (%)	Contribution to CS rate of 15.8% (pp)	n in Robson Group	% of Small Business in Robson Group	C-section rate in Robson Group (%)	Contribution to CS rate of 18.4% (pp)	n in Robson Group	% of Professional in Robson Group	C-section rate in Robson Group (%)	Contribution to CS rate of 21.1% (pp)	n in Robson Group	% of students in Robson group	C-section rate in Robson Group (%)	Contribution to CS rate of 13.8% (pp)
1&2	3757	31	13	4.1	1962	28	13	3.6	542	25	14	3.4	407	31	17	5.3	746	54	13	6.8
3&4	3308	27	7	1.9	2082	29	6	1.9	693	32	8	2.6	401	31	9	2.6	85	6	6	0.4
5	382	3	100	3.1	223	3	100	3.1	74	3	100	3.4	70	5	100	5.4	8	1	100	0.6
6	98	1	89	0.7	49	1	84	0.6	17	1	94	0.7	16	1	100	1.2	15	1	93	1.0
7	88	1	85	0.6	52	1	89	0.6	22	1	77	0.8	11	1	82	0.7	1	0	100	0.1
8	228	2	45	0.8	143	2	43	0.9	42	2	52	1.0	21	2	38	0.6	17	1	47	0.6
9	69	1	90	0.5	38	1	90	0.5	14	1	100	0.6	6	1	100	0.5	10	1	70	0.5
10	1163	10	15	1.4	714	10	14	1.4	203	9	17	1.6	84	6	14	0.9	144	11	13	1.4
11	1223	10	12	1.2	655	9	12	1.1	158	7	15	1.1	104	8	12	0.9	280	20	10	2.0
12	1622	13	14	1.9	1058	15	12	1.8	352	16	18	2.9	155	12	21	2.5	32	2	13	0.3
missing^c^	271	2	13	0.3	153	2	13	0.3	44	2	9	0.2	29	2	24	0.5	37	3	8	0.2
Total	12,209	100	17	16.5	7129	100	16	15.8	2161	100	18	18.4	1304	100	21	21.1	1375	100	14	13.8

^a^Group:

1&2: Nullipara, singleton cephalic, 37+ weeks, spontaneous & induced labour

3&4: Multipara (excluding previous C-section) singleton cephalic, 37+ weeks, spontaneous & induced labour

5: Previous caesarean section, singleton cephalic, 37+ weeks

6: All nulliparous breeches

7: All multiparous breeches (including previous C-section)

8: All multiple pregnancies (including previous C-section)

9: All abnormal lies (including previous C-section but excluding breech)

10: All singleton cephalic, < 37 weeks (including previous C-section)

11: All nullipara, singleton cephalic, gestational age unknown (newly developed category)

12: All multipara, singleton cephalic, gestational age unknown (newly developed category)

^b^pp.: percentage point; ^c^ 271 women could not be divided in one of the Robson groups (also not in newly developed category 11 or 12), because of missing information on parity (16 records), number of fetuses (236 records) or a combination of missing parity and number of fetuses (19 records)

Also, when only considering women without a previous C-section, C-section rates were higher among professionals (14.5%) than among other groups (housewives: 11.5%, students 13.1%) (Figure S1A), although these differences were smaller than in the total study population. Among women without previous C-section, the prevalence of indication was somewhat higher among professionals (9%) than among housewives (7%), which was for a large part explained by age and parity (Figure S1B-C). 43% of C-sections among women without previous C-section were not medically indicated (Figure S1D); this was similar (42% [13/31]) in our review of manual patient files. Almost one third of the three pp. difference in C-section rate between professionals and housewives was due to medically non-indicated C-sections (Figure S1B). The higher odds of C-section among professionals compared with housewives (OR 1.3; 95%CI 1.09–1.56) was largely explained by the combination of indication, age and parity (aOR 1.03; 95%CI 0.83–1.28) (Figure S1E).

## Discussion

### Main findings

Our study shows that unnecessary primary C-sections and near universal repeat C-sections play an important role in explaining both the overall C-section rate and socioeconomic inequalities in C-section. Socioeconomic inequalities in C-section were moderate in the Kenyan referral hospital that we studied. These inequalities were foremost explained by a higher level of indicated C-sections -mostly related to previous C-section- among high SEP women. Nearly all women with a previous C-section had a repeat C-section for their subsequent pregnancy, and 3 in 10 C-sections had previous C-section as indication. But over-use of C-sections based on hospital guidelines was also substantial, and seen in all socioeconomic groups: over 4 in 10 primary C-sections had no documented indication. Higher over-use among high SEP women explained around one third of socioeconomic inequalities in primary C-sections. Socioeconomic differences in age and parity further contributed to explaining inequalities in indicated and unindicated C-sections. Our study suggests that prevention of unnecessary primary C-sections and promotion of safe trial of labor with close monitoring in women with a scarred uterus could help curb the C-section epidemic and help reduce socioeconomic differences in C-section.

### Strengths and limitations

Our analyses suffered from some problems. First, we used anonymised versions of the Delivery and Surgery Databases, which complicated patient identification and linking of the databases due to typos in patient numbers. 286 C-section records in the Delivery Database (2% of all deliveries, 13.2% of C-section deliveries) could not be matched to a Surgery Database record, and 148 C-section records in the Surgery Database (1% of all deliveries, 6.8% of C-sections) could not be matched to a Delivery Database record. To avoid over-estimating the C-section rate, we used the Delivery Database as basis for our analyses, rather than including all unlinked records. If we also had included the 148 unlinked records from the Surgery Database, the C-section rate would have been 17.1% instead of 16.5%.

Secondly, the analyses suffered from some uncertainty in determining clinical indication for C-section because only a limited set of variables was available in the electronic databases. Our use of the precautionary principle, as explained in the methods section, will probably have led to an overestimation of the proportion of caesarean deliveries with an indication. Importantly, multiple previous C-sections constituted a C-section indication according to the hospital guidelines, while information on the number of previous C-sections missed in the electronic records. Use of the precautionary principle led to the classification of all previous C-sections as indication, while many will have been first repeats. Also, we were not able to take into account clinical judgement not recorded in the electronic database. This may have led both to an under-estimation or over-estimation of the proportion of caesarean deliveries with a clinical indication. Detailed analysis of a random selection of the full manual files of C-section patients confirms our estimate of C-section over-use. Furthermore, there is no indication that an over- or underestimation of clinical indication for C-section would be differential by SEP.

Finally, maternal occupation as recorded in the patient files is a rough proxy for SEP, arguably with measurement error both in determining occupation itself and in classifying occupation into categories. There is no indication that such measurement error was systematic. Combined with the broad occupational categories used, random measurement error in occupational class will have led to an underestimation of socioeconomic differences in C-section rate.

### Generalizability

Our findings pertain to an academic referral hospital and are not generalizable to Kenya at large, where nearly 40% of women have home births and, consequently, C-section rates at the population level are lower [4]. Socioeconomic differences in C-section rates are much larger in Kenya at large, as they partly capture socioeconomic differences in facility birth. Yet, the C-section rate in our study hospital was comparable to the institutional C-section rate in Kenya as a whole [4]. Given that the hospital draws on a broad catchment population, and that only a tiny proportion of women used the hospital as referral hospital, one might see our findings as a precursor of what may happen in the rest of Kenya -and arguably other low and middle income countries- when facility birth rates increase further, especially when repeat C-sections are highly common. At the same time, the C-section rate in our study hospital was still modest compared with those observed in some countries where population-level C-section rates reach up to 40–60% [30]. In such countries, the contribution of unnecessary primary C-sections to (inequalities in) the C-section rate will be much larger than in our study.

### Research implications

First, our study shows that a combination of criterion-based auditing and equity analysis can help gain a better understanding of drivers of C-section rates and inequalities in these rates – a first step to curb increasing over-use. Our study of over 12,000 births was only practically feasible because of the availability of electronic patient records. Electronic records can facilitate monitoring, and our study shows the potential for using hospital record data for improvements in health care delivery. At the same time, a more detailed documentation of decisions around mode of birth, including if C-section was on demand, is advisable for accountability purposes and to improve quality of care. Second, our study shows that socioeconomic differences in C-section rates, especially in contexts of moderate C-section rates and near universal repeat C-sections, can be largely explained by differences in medical indication (largely due to previous C-section), age and parity. This should be taken into account in future explanatory research on socioeconomic differences in C-section rates. Third, qualitative research on decisions around primary C-sections, both in the context of moderate C-section rates as in Kenya, as in the context of very high C-section rates such as for example Colombia, will be important to understand demand and supply side mechanisms that drive over-use. Finally, future research should address the paucity of evidence on how to safely and effectively reduce primary and repeat C-section rates in resource poor countries [31].

### Policy implications

Our findings suggest that unnecessary primary C-sections, combined with a practice of near universal repeat C-sections fuel the C-section epidemic. Unnecessary primary C-sections cause needless maternal and infant morbidity [5–8, 32]. The incidence of uterus rupture in women with a prior C-section, for example, is 1% in resource-poor countries [33]. Unnecessary primary C-sections combined with near universal repeat C-sections lead to a cascade of C-sections. Our finding that repeat C-sections substantially contribute to (inequalities in) the C-section rate correspond Vogel et al.’s conclusions that repeat C-section are an increasingly important driver of C-section rates in low- and middle-income countries [34]. We add that they are also an important driver of socioeconomic inequalities in C-section rates.

Little is known about how to effectively reduce unnecessary primary C-sections in low and middle income countries [35, 36]. Some evidence suggests that audit and feedback can reduce C-section rates [37] and that this is feasible in Sub-Saharan African contexts [38]. Changes in financial incentives for hospitals and doctors in combination with better pain relief and support during labor may also be effective [39]. Furthermore, investments in training and equipment for assisted vaginal birth, especially vacuum extraction, can reduce C-section rates in case of prolonged second stage labor or foetal distress [40]. While assisted vaginal birth is associated with reductions in morbidity and mortality, especially in resource-poor countries [40], such births remain rare in these settings [41]. Promoting safe trial of labor with close monitoring in women with a scarred uterus can reduce the prevalence of repeat C-sections, although high-quality evidence on the benefits and harms of vaginal birth after C-section remains scarce [42, 43]. Prevention of unnecessary primary C-sections and promoting safe trial of labor should be part of broader efforts to improve quality of maternity care, which should include shared decision making [44].

## Conclusions

Higher C-section rates among better-off women can be partly explained by unnecessary primary C-sections and by higher supposed medical need due to previous C-section. Prevention of unnecessary primary C-sections and promoting safe trial of labor with close monitoring in women with a scarred uterus should be a priority in addressing over-use of C-section.

## Supplementary information

**Additional file 1: Table S1.** Main indication, sub-indications and information required to judge clinical indication for C-section. Main indications and required information are displayed in ICD-codes (ICD-10). **Table S2.** The odds of medical indication for C-section according to the hospital guidelines (Students instead of Housewives as reference category). **Table S3**. Mode of delivery according to indication for C-section. **Table S4.** Socioeconomic inequalities (measured in odds ratios) in C-section rate, without and with adjustment for clinical indication for C-section, previous C-section, multiple birth, presentation, gestational age, maternal age, and parity (Students compared with other socioeconomic groups). **Figure S1.** Women without previous C-section.

## Data Availability

The data that support the findings of this study are available from the study hospital but restrictions apply to the availability of these data, and so are not publicly available. Data are however available from the authors upon reasonable request and with permission of the study hospital.
