# Supplementary material for: Socioeconomic differences in caesarean section – are they explained by medical need? An analysis of patient record data of a large Kenyan hospital
Source: Int J Equity Health. 2020 Jul 8;19:117. doi: 10.1186/s12939-020-01215-2 (PMC7341621; doi:10.1186/s12939-020-01215-2)
Supplement: Supplementary file 1 — Additional file 1: Table S1. Main indication, sub-indications and information required to judge clinical indication for C-section. Main indications and required information are displayed in ICD-codes (ICD-10). Table S2. The odds of medical indication for C-section according to the hospital guidelines (Students instead of Housewives as reference category). Table S3. Mode of delivery according to indication for C-section. Table S4. Socioeconomic inequalities (measured in odds ratios) in C-section rate, without and with adjustment for clinical indication for C-section, previous C-section, multiple birth, presentation, gestational age, maternal age, and parity (Students compared with other socioeconomic groups). Figure S1. Women without previous C-section. [file 12939_2020_1215_MOESM1_ESM.docx]

# Table S1: Main indication, sub-indications and information required to judge clinical indication for C-section. Main indications and required information are displayed in ICD-codes (ICD-10)

| **Main indication plus ICD-10 codes** | **Sub-indication per guideline ^a b c^** | **Information needed** |
| --- | --- | --- |
| Presentation & position | | |
| Breech presentation | **Local hospital guidelines** | |
| O32.1 (Breech presentation)  O64.1 (Obstructed labour due to breech presentation)  O80.1 (Spontaneous breech delivery)  O83.0 (Breech extraction)  O83.1 (Breech delivery NOS) | Cord presentation | O69.0 (Labour and delivery complicated by prolapse of cord) |
|  | Foetal growth restriction | O36.5 (Maternal care for poor foetal growth)  P05.9 (Slow foetal growth, foetal growth retardation NOS) ^d^ |
|  | Macrosomia | O33.5 (Maternal care large foetus)  O36.6 (Maternal care for excessive foetal growth)  O66.2 (Obstructed labour due to large foetus) ^d^ |
|  | Foetal anomaly incompatible with spontaneous vertex delivery (SVD) | Compatibility with SVD ^e^  O35.9 (Foetal abnormality (malformed) and damage) ^d^ |
|  | Previous C-section | O34.2 (Maternal care due to uterine scar from previous surgery) |
|  | **Kenya** | |
|  | Double footling breech | Double footling breech ^e^ |
|  | Small foetus | O36.5 (Maternal care for poor foetal growth)  P05.9 (Slow foetal growth, foetal growth retardation NOS) ^d^ |
|  | Malformed foetus | O35.9 (Foetal abnormality (malformed) and damage) ^d^ |
|  | Macrosomia | O33.5 (Maternal care large foetus)  O36.6 (Maternal care for excessive foetal growth)  O66.2 (Obstructed labour due to large foetus) ^d^ |
|  | Previous C-section | O34.2 (Maternal care due to uterine scar from previous surgery) |
|  | Myomectomy | 5-681 (Excision or destruction of lesion of uterus; endometrectomy, myomectomy, division of endometrial synechiae) ^d^ |
|  | Hyper extended/deflexed head | Information about hyper extended/deflexed head ^e^  O64.0 (Obstructed labour due to incomplete rotation of foetal head) |
|  | **The Netherlands** | |
|  | For whom external cephalic version is contraindicated or has been unsuccessful. | Whether version was (unsuccessfully) tried ^e^ |
|  | **England** | |
|  | For whom external cephalic version is contraindicated or has been unsuccessful. | Whether version was (unsuccessfully) tried ^e^ |
|  | Previous C-section | O34.2 (Maternal care due to uterine scar from previous surgery) |
|  | Ruptured membranes | Ruptured membranes ^e^ |
|  | Foetal compromise | O68 or O68.8 or O68.9 (Foetal stress) |
|  | Vaginal bleeding | N93.9 or N93.8 or N93 (Vaginal bleeding) ^d^ |
|  | Other medical condition | Z87 (Personal history of diseases and conditions) |
| Compound presentation | **Kenya** | |
| O64.5 (Obstructed labour due to compound presentation) ^d^  O32.6 (Maternal care for compound presentation) | Attempt of pushing back the arm of the baby fails | Information about attempt to push the baby back ^e^ |
|  | Cord prolapses | O69.0 (Labour and delivery complicated by prolapse of cord) |
|  | Other complications occur | Information about complications ^e^ |
| Malposition | **Kenya** | |
|  | Face presentation mento-posterior position | O64.2 (Obstructed labour due to face and chin presentation) O32.3 (Maternal care for chin, face, brow presentation) O64.8 (Obstructed labour due to other malposition and malpresentation) ^d^ |
|  | Brow presentation | O64.3 (Obstructed labour due to brow presentation) ^d^  O32.3 (Maternal care for chin, face, brow presentation)  O64.8 (Obstructed labour due to other malposition and malpresentation) ^d^ |
|  | Occiput posterior Position if there are signs of obstruction or foetal heart rate is abnormal. | Abnormality of foetal heart rate ^e^  O64.0 (Obstructed labour due to incomplete rotation of foetal head incl occipito-posterior)  O64.8 (Obstructed labour due to other malposition and malpresentation) ^d^ |
|  | Occiput-transverse position if Kielland’s forceps and/ or vacuum extraction fail | Forceps and/or vacuum being tried unsuccessfully ^e^  O64.0 (Obstructed labour due to incomplete rotation of foetal head incl occipito-transverse) O64.8 (Obstructed labour due to other malposition and malpresentation) ^d^ |
| Transverse lie | **Kenya** | |
| O32.2 (Maternal care for transverse and oblique lie)  O64 (Obstructed labour due to malposition and malpresentation foetus) ^d^  O64.9 (Obstructed labour due to malposition and malpresentation foetus) | When external version (if the woman is in early labour and the membranes are intact) fails | Whether version was (unsuccessfully) tried ^e^  Information about the membranes ^e^  Progression of labour (partogram) ^e^ |
|  | **The Netherlands** | |
|  | At term pregnancy | Gestation |
| Multiple gestation | | |
| Twin pregnancy | **Local hospital guidelines** | |
| O30.0 (Twin pregnancy) | First twin transverse/oblique lie | Presentation of individual foetuses ^e^  O32.2 (Transverse and oblique lie)  O32.5 (Multiple gestation with malpresentation of one foetus or more) O64.4 (Obstructed labour due to shoulder presentation, incl. prolapsed arm) O64.8 (Obstructed labour due to other malposition and malpresentation) ^d^ |
|  | First twin breech (EL C-section or vaginal delivery) | Presentation of individual foetuses ^e^  O32.1 (Breech presentation)  O32.5 (multiple gestation with malpresentation of one foetus or more) O64.1 (Obstructed labour due to breech presentation)  O80.1 (Spontaneous breech delivery) O83.0 (Breech extraction)  O83.1 (Breech delivery NOS) |
|  | First twin cephalic while second twin transverse when podalic version fails | Whether version was (unsuccessfully) tried ^e^  Presentation of individual foetuses  O32.5 (multiple gestation with malpresentation of one foetus or more) |
|  | **Kenya** | |
|  | First twin not cephalic | Presentation of individual foetuses ^e^  O32.1, O64.1, O80.1, O32.2, O32.5, O83.0, O83.1 (Presentation other than cephalic) |
|  | Retained second twin (>30 min after delivery of first twin) | Time of retained 2nd twin ^e^  O63.2 (Delayed delivery of second twin/triplet) ^d^ |
|  | Extreme prematurity | P07.3 (Other preterm infants; 28 completed weeks or more but less than 37 weeks) ^d^ |
|  | Discordant foetal growth | Information about discordant foetal growth ^e^ |
|  | Mono-amniotic sac | O30.01 (Mono-amniotic twin pregnancy) |
|  | Conjoined twins | Q89.4 (Conjoined twins) ^d^ O33.7 (Maternal care for disproportion due to other foetal deformities, including: conjoined twins, ascites, hydrops, tumour, meningomyelocele; causing disproportion) ^d^  O66.3 (Obstructed labour due to other abnormalities of foetus (including: conjoined twins)) |
|  | **The Netherlands** | |
|  | Mono-amniotic sac | O30.01 (Mono-amniotic twin pregnancy) ^d^ |
|  | First twin breech for which external cephalic version is contraindicated or has been unsuccessful. | Presentation of individual foetuses ^e^  Whether version was (unsuccessfully) tried ^e^  O32.1 (Breech presentation) O64.1 (Obstructed labour due to breech presentation) O80.1 (Spontaneous breech delivery) O83.0 (Breech extraction) O83.1 (Breech delivery NOS) O32.5 (Multiple gestation with malpresentation of one foetus or more) |
|  | **England** | |
|  | First twin not cephalic | Presentation of individual foetuses ^e^  O32.1, O64.1, O80.1, O32.2, O32.5, O83.0, O83.1 (Presentation other than cephalic) |
| Other multiple gestation | **Local hospital guidelines** | |
|  | More than two foetuses if foetal survival is expected | Foetal survival is expected yes or no ^e^  O30.1 (Triplets) O30.2 (Quadruplet) |
|  | **Kenya** | |
|  | More than two foetuses | O30.1 (Triplets) O30.2 (Quadruplet) |
| Previous Caesarean Section | | |
| Previous scar | **Local hospital guidelines** | |
| O34.2 (Maternal care due to uterine scar from previous surgery)  O75.7 (Vaginal delivery following previous C-section,  TOLAC) ^d^ | two or more previous C-section | Amount of previous C-section ^e^ |
|  | BMI > 30 | Z68.30 (BMI 30-30.9) ^d^ |
|  | Woman declines trial of labour after C-section (TOLAC) | Woman refusal for TOLAC yes or no ^e^ |
|  | Lack of progress from TOLAC in two hrs despite adequate contractions (and augmentation) | Progress of labour (partogram) ^e^  O75.7 (vaginal delivery following previous C-section, TOLAC) ^d^ O66.4 (Failed trial of labour, unspecified) |
|  | Non reassuring foetal status during TOLAC | O75.7 (vaginal delivery following previous C-section, TOLAC) ^d^ O66.4 (Failed trial of labour, unspecified) |
|  | Transverse lie in previous C-section | Presentation in previous C-section ^e^ |
|  | Scar <18 months old | Age of scar ^e^ |
|  | **Kenya** | |
|  | Two or more previous C-section | Amount of previous C-section ^e^ |
|  | Vertical incision in previous C-section | Incision in previous C-section ^e^ |
|  | Uterus rupture in previous birth | Uterine rupture in the past ^e^  Z35.2 (supervision of pregnancy with other poor reproductive or obstetric history; conditions (O10-O92), neonatal death, stillbirth) ^d^ |
|  | **The Netherlands** | |
|  | Three or more previous C-section | Amount of previous C-section ^e^ |
|  | Uterus rupture in previous birth | Uterine rupture in the past (only bad obstetric history)  Z35.2 (supervision of pregnancy with other poor reproductive or obstetric history; conditions (O10-O92), neonatal death, stillbirth). ^d^ |
|  | Vertical incision in previous C-section | Incision in previous C-section ^e^ |
|  | **England** | |
|  | Uterus rupture in previous birth | Uterine rupture in the past ^e^  Z35.2 (supervision of pregnancy with other poor reproductive or obstetric history; conditions (O10-O92), neonatal death, stillbirth). ^d^ |
|  | Vertical incision in previous C-section | Incision in previous C-section ^e^ |
| Placenta and cord problems | | |
| Placenta praevia | **Kenya** | |
| O44.0 (Placenta praevia without haemorrhage) ^d^  O44.1 (Placenta praevia, marginal, partial or total with haemorrhage) | Major placenta praevia  Type IIb-IV | Location of placenta ^e^  Type of placenta praevia ^e^ |
|  | **The Netherlands** | |
|  | Placenta is located <10mm from the cervical os (total and lateral placenta praevia) | Distance to cervical os ^e^  Location of placenta ^e^ |
|  | Blood loss cannot be compensated | Compensation of blood loss yes or no ^e^ |
|  | **England** | |
|  | Placenta partly or completely covers the cervical os (minor or major placenta praevia) | Distance to cervical os ^e^  Location of placenta ^e^ |
|  | Placenta is located <20mm from the cervical os in the third trimester | Distance to cervical os ^e^  Location of placenta ^e^ |
| Morbidly adherent | **England** | |
| placenta  O43.2 (Morbidly adherent placenta; accrete, increta, percreta) ^d^ | -^f^ | - |
| Vasa praevia | **Local hospital guidelines** | |
| O69.4 (Labour and delivery complicated by vasa praevia Incl.: Haemorrhage from vasa praevia) ^d^ | - ^f^ | - |
|  | **Kenya** | |
|  | - ^f^ | - |
|  | **The Netherlands** | |
|  | - ^f^ | - |
|  | **England** | |
|  | Bleeding vasa praevia | Bleeding yes or no ^e^ |
|  | Term pregnancy | Gestation |
| Cord prolapse | **Local hospital guidelines** | |
| O69.0 (Labour and delivery complicated by prolapse of cord) | Foetal heart tone is heard | Foetal heart tone yes or no ^e^ |
|  | **Kenya** | |
|  | First stage of labour | Progress of labour (partogram) ^e^ |
|  | Second stage of labour when in doubt about pelvic capacity | Progress of labour (partogram) ^e^  Pelvic capacity ^e^ |
|  | **England** | |
|  | - ^f^ |  |
| Foetal distress | | |
| Foetal distress | **Local hospital guidelines** | |
| O68 (Labour and delivery complicated by foetal stress (distress  O68.9 (Labour and delivery complicated by foetal stress, unspecified)  O68.8 (Foetal stress evidenced by electrocardiographic, ultrasonic) | - ^f^ |  |
|  | **Kenya** | |
|  | When the foetal heartrate is still abnormal after: changing position of mother, stop oxytocin, hydrate with IV dextrose 5% (1liter in 30 min), giving oxygen to the mother. | Information about changing position of mother, giving oxygen, giving IV dextrose 5% ^e^  P20 (intrauterine hypoxia incl abnormal foetal heart rate)^d^  P20.0 (Intrauterine hypoxia first noted before onset of labour) ^d^  P20.1 (Intrauterine hypoxia first noted during labour and delivery) ^d^  P20.9 (Intrauterine hypoxia, unspecified) ^d^ |
|  | **The Netherlands** | |
|  | **-** ^f^ | - |
|  | **England** | |
|  | - ^f^ | - |
| Foetal anomalies | | |
| Hydrocephalus | **Kenya** | |
| O33.6 (Maternal care for disproportion due to hydrocephalic)  O66.3 (Obstructed labour due to other abnormalities of foetus; foetal ascites, hydrops, meningomyelocele, sacral teratoma, tumour, hydrocephalic foetus) | - ^f^ |  |
| Sacral tumour foetus | **Kenya** | |
| O66.3 (Obstructed labour due to other abnormalities of foetus; foetal ascites, hydrops, meningomyelocele, sacral teratoma, tumour, hydrocephalic foetus) | - ^f^ |  |
| Maternal diseases | | |
| Pre-eclampsia | **Local hospital guidelines** | |
| O14.0 (Mild to moderate pre-eclampsia) O14.1 (Severe pre-eclampsia)  O14.9 (pre-eclampsia, unspecified) | Foetal distress | O68 (labour and delivery complicated by foetal stress (distress) O68.9 (labour and delivery complicated by foetal stress, unspecified)  O68.8 (Foetal stress evidenced by electrocardiographic , ultrasonic) |
|  | Arrest of dilatation | O62.0 (primary inadequate contractions; failure of cervical dilatation) |
|  | **Kenya** | |
|  | Severe pre-eclampsia | O14.1 (Severe pre-eclampsia) |
|  | Unfavourable cervix | O62.0 (primary inadequate contractions; failure of cervical dilatation) |
| Impending uterus rupture | **Local hospital guidelines** | |
| O71.0 (Rupture of uterus before onset of labour)  O71.1 (Rupture of uterus during labour) | - ^f^ | - |
|  | **England** | |
|  | - ^f^ | - |
| Big cervical myomas | **The Netherlands** | |
| O34.4 (Abnormalities of cervix, including Cervical polyp)  D25.9 (Leiomyoom) ^d^ | - ^f^ | - |
| Pelvic tumour obstructing | **Kenya** | |
| labour  O65.8 (Obstructed labour due to other maternal pelvic abnormalities) ^d^  O34.1 (Tumour of corpus uteri)  O34.4 (Abnormalities of cervix, including tumour)  R19.0 (Intra-abdominal and pelvic swelling, mass and lump) | - ^f^ | - |
| Invasive carcinoma of the | **Kenya** | |
| cervix  O34.4 (Maternal care for other abnormalities of cervix, including tumour, polyp, stricture/stenosis, previous surgery to cervix)  D06 (Carcinoma in situ of cervix uteri) ^d^ | - ^f^ |  |
| Delivery following repair of obstetric fistula | **Kenya** | |
| There is no ICD code for delivery **following** repair of obstetric fistula, and therefore could not be included in our analysis. | - ^f^ |  |
| Contracted pelvis | **Kenya** | |
| O65.1 (Obstructed labour due to generally contracted pelvis) ^d^ | - ^f^ |  |
| Medical conditions | **Local hospital guidelines** | |
|  | Comatose very ill patient | R40 (Somnolence, stupor and coma) ^d^ R40.0 (Somnolence) ^d^ R40.1 (Stupor) ^d^ R40.2 (Coma, unspecified) ^d^ |
|  | **Kenya** | |
|  | Severe heart disease | I11.0 (Hypertensive heart disease with heart failure) ^d^ I11.9 (Hypertensive heart disease without heart failure) ^d^ I50.9 (Heart failure, unspecified) |
|  | Severe respiratory disease | O99.5 (Disease of respiratory system complicating pregnancy, childbirth, puerperium) |
|  | Severe hypertension | O10.0 (Pre-existing essential hypertension complicating pregnancy, childbirth, puerperium) O10.1 (Pre-existing hypertensive heart disease complicating pregnancy, childbirth, puerperium)^d^ O16 (Unspecified maternal hypertension) |
|  | Cerebral aneurysm | I67.1 (Cerebral aneurysm, non-ruptured) ^d^ |
|  | Musculoskeletal disorders | M96 (Post procedural musculoskeletal disorders, not elsewhere classified) ^d^ |
|  | Severe neurological disorders | R29 (Other symptoms and signs involving the nervous and musculoskeletal systems) ) ^d^ |
| Maternal infections | | |
| Hepatitis B | **Kenya** | |
| B16 Acute hep B ^d^  B16.0/B16.1 (Acute hep B with delta-agent with/without hepatic coma) ^d^  B16.2/B16.9 (Acute hep B without delta-agent with/without hepatic coma) ^d^  B18.0 (Chronic viral hep B with delta-agent) ^d^  B18.9 (Chronic viral hep B without delta-agent) ^d^ | - ^f^ | - |
| Primary genital Herpes Simplex Virus | **Local hospital guidelines** | |
| B07 (Viral warts: simplex, vulgaris)  B00 (Herpes viral (herpes simplex) infections)^d^ | - ^f^ | - |
|  | **Kenya** | |
|  | - ^f^ | - |
|  | **The Netherlands** | |
|  | - ^f^ | **-** |
|  | **England** | |
|  | Virus occurring in 3^rd^ trimester | Which trimester the virus occurred ^e^ |
| HIV | **Kenya** | |
| B24 (Unspecified human immunodeficiency virus disease HIV)  B21.0 (HIV disease resulting in Kaposi sarcoma)  B20-B24 (HIV specified) ^d^ | Viral load of >1000 copies per ml | Viral load ^e^ |
|  | **The Netherlands** | |
|  | Viral load 50 or more copies per ml | Viral load ^e^ |
|  | Not on HAART therapy | (What) treatment  ^e^ |
|  | **England** | |
|  | Not on treatment with any anti-retroviral therapy and a viral load of 50 or more copies per ml | Viral load ^e^  (What) treatment  ^e^ |
|  | Not on HAART therapy and a viral load of 400 or more copies per ml | Viral load ^e^  (What) treatment  ^e^ |
| HIV + Hepatitis C Virus | **England** | |
| B24 (Unspecified human immunodeficiency virus disease HIV) or  B21.0 (HIV disease resulting in Kaposi sarcoma) + O98.4 (viral hepatitis complicating pregnancy, childbirth and the puerperium) (B15-B19 including) or  B20-B24 (HIV specified)  +  O98.4 (Viral hepatitis complicating pregnancy, childbirth and the puerperium) (B15-B19 including) ^d^ | - ^f^ | - |
| Other indications | | |
| Antepartum haemorrhage | **Local hospital guidelines** | |
| O46 (APH, not elsewhere classified) ^d^  O46.0 (APH with coagulation defect)  O46.8 (Other APH)  O46.9 (APH, unspecified) | Unstable woman | Mother stable or not ^e^ |
|  | **Kenya** | |
|  | Poor progress of labour | O63 (Long labour) ^d^ O63.0 (Prolonged first stage) ^d^ O63.1 (Prolonged second stage)  O63.9 (Long labour, unspecified) |
|  | Failure of uterus to relax between  contractions | O62.4 (Hypertonic, in coordinate, and prolonged uterine contractions) |
|  | Foetal distress | O68 (Labour and delivery complicated by foetal stress (distress) O68.9 (Labour and delivery complicated by foetal stress, unspecified)  O68.8 (Foetal stress evidenced by electrocardiographic , ultrasonic) |
|  | Increased bleeding | Intensity of the bleeding over time ^e^ |
|  | **England** | |
|  | Foetal distress and stable mother | Mother stable or not ^e^  O68 (Labour and delivery complicated by foetal stress (distress)  O68.9 (Labour and delivery complicated by foetal stress, unspecified)  O68.8 (Foetal stress evidenced by electrocardiographic , ultrasonic) |
| Obstructed labour | **Local hospital guidelines** | |
|  | Large foetus | O65.4 (Obstructed labour due to fetopelvic disproportion)  O66.2 (Obstructed labour due to large foetus) ^d^  O66.9 (Obstructed labour, unspecified)  +  O33.5 (Maternal care for disproportion due to unusually large foetus) or  O36.6 (Maternal care for excessive foetal growth) |
|  | **Kenya** | |
|  | Cephalo-pelvic disproportion is confirmed | O33.9 (Maternal care for disproportion, unspecified; cephalo-pelvic disproportion NOS, feto-pelvic disproportion NOS)  O65.4 (Obstructed labour due to feto-pelvic disproportion, unspecified) O65.9 (Obstructed labour due to maternal pelvic abnormality, unspecified) O65 (Obstructed labour due to maternal pelvic abnormality) ^d^ O65.1/O65.2/O65.3/O65.5/O65.8 (All obstructed labour due to maternal pelvic abnormality specified) ^d^ |
|  | Foetus is alive, cervix is not fully dilated | Foetus alive yes or no ^e^  O62.0 (Primary inadequate contractions; failure of cervical dilatation) |
|  | Foetus is alive but the foetal head is too high for vacuum extraction | Foetus alive yes or no ^e^  O32.4 (Maternal care for high head at term (failure of head to enter pelvic brim)) |
| Post term | **Local hospital guidelines** | |
| O48 (Prolonged pregnancy; post-dates, post-term) | >42 weeks and at least one previous C-section | Gestation  O34.2 (Maternal care due to uterine scar from previous surgery) |
| Prolonged labour | **Local hospital guidelines** | |
| O63 (Long labour) ^d^  O63.0 (Prolonged first stage) ^d^  O63.1 (Prolonged second stage)  O63.9 (Long labour, unspecified) | Delivery is not imminent after 60 min of active pushing | Progress of labour (partogram) ^e^ |
|  | **Kenya** | |
|  | The foetal head is more 3/5 above the symphysis after augmentation in 2^nd^ stage | Progress of labour (partogram) ^e^ |
|  | Leading bony edge of the foetal head is above -2 station after augmentation in 2^nd^ stage | Induction of labour yes or no ^e^ |
|  | **The Netherlands** | |
|  | Dilation of <1-2 cm an hour | Progress of labour (partogram) ^e^ |
|  | Expulsion of >30-60 min | Progress of labour (partogram) ^e^ |
|  | **England** | |
|  | Dilation of <2cm after 4 hours of oxytocin | Progress of labour (partogram) ^e^  Induction of labour yes or no ^e^ |

^a^ England: The RCOG guidelines are applicable to England and Wales, the NICE guidelines are applicable to England.

^b^ Shoulder dystocia is an indication for C-section according to the Kenyan and Dutch guidelines. We decided, however, not to include this as an indication for C-section in our study, because, at this stage of labour, the head of the baby is almost born. In order to perform a C-section, the head of the baby would need to be pushed back into the mother. This has been theoretical described (Zavanelli’s manoeuvre), but practically never done.

^c^ An indication according to the Kenyan guideline included delivery following repair of obstetric fistula. As there is no ICD code for this indication, we could not implement this indication in our analysis.

^d^ These codes exist according to the ICD-10, but they were not reported in the Delivery Database.

^e^ This information is not documented in the Delivery Database and therefore this information is missing.

^f^ There is no sub-indication for a C-section mentioned in this guideline, only a main indication (for example: vasa praevia is, by itself, an indication for C-section according to the local hospital guidelines).

**Table S2:** The odds of medical indication for C-section according to the hospital guidelines (Students instead of Housewives as reference category)

|  | **Univariate** | | **Adjusted for maternal age ^a^** | | **Adjusted for Parity ^b^** | | **Adjusted for previous C-section** | | **Adjusted for all ^a b^** | |
| --- | --- | --- | --- | --- | --- | --- | --- | --- | --- | --- |
|  | OR [95% CI] | P value | OR[95% CI] | P Value | OR[95% CI] | P Value | OR [95% CI] | P Value | OR [95% CI] | P Value |
|  |  |  |  |  |  |  |  |  |  |  |
| *Occupation* |  |  |  |  |  |  |  |  |  |  |
| Housewife | 1.35 (1.10;1.66) | 0.0035 | 1.07 (0.87;1.32) | 0.5087 | 1.24 (1.01;1.53) | 0.0411 | 0.84 (0.68;1.05) | 0.1294 | 0.84 (0.67;1.06) | 0.1402 |
| Small business | 1.7 (1.36;2.13) | 0.0000 | 1.24 (0.98;1.58) | 0.0693 | 1.54 (1.23;1.95) | 0.0002 | 1.02 (0.79;1.31) | 0.8935 | 0.98 (0.75;1.29) | 0.9054 |
| Professional | 2.01 (1.58;2.55) | 0.0000 | 1.41 (1.09;1.81) | 0.0079 | 1.86 (1.46;2.37) | 0.0000 | 1.12 (0.85;1.49) | 0.4204 | 0.99 (0.73;1.33) | 0.9419 |
| Student | 1 |  | 1 |  | 1 |  | 1 |  | 1 |  |

a adjustment for age in years; b adjustment for parity in actual number of births (not in parity categories)

# Table S3: Mode of delivery according to indication for C-section

|  | **Total population** | **Housewife** | **Small business** | **Professional** | **Student** |
| --- | --- | --- | --- | --- | --- |
| Total | 12,209 | 7,129 | 2,161 | 1,304 | 1,375 |
|  |  |  |  |  |  |
| *Local hospital guidelines* |  |  |  |  |  |
| % of women with C-section indication | 11.7 | 11.2 | 13.7 | 15.7 | 8.5 |
| C-section rate among women with indication | 98.0 | 97.6 | 98.6 | 99.0 | 98.3 |
| C-section rate among women without indication | 5.7 | 5.5 | 5.7 | 6.6 | 6.0 |
| Among C-section deliveries: % without  indication | 30.5 | 30.8 | 26.9 | 26.2 | 39.5 |
| Unmet need (among women with indication: % without CS) | 2.0 | 2.4 | 1.4 | 1.0 | 1.7 |
| Among all deliveries: % with CS with indication | 11.5 | 10.9 | 13.5 | 15.6 | 8.4 |
| Among all deliveries: % with CS without indication | 5.0 | 4.9 | 5.0 | 5.5 | 5.5 |
| Among all deliveries: % with vaginal delivery with CS indication | 0.2 | 0.3 | 0.2 | 0.2 | 0.1 |
|  |  |  |  |  |  |
| *Kenyan guidelines* |  |  |  |  |  |
| % of women with C-section indication | 14.0 | 13.2 | 15.9 | 18.4 | 11.2 |
| C-section rate among women with indication | 94.0 | 94.0 | 93.9 | 94.6 | 94.2 |
| C-section rate among women without indication | 4.0 | 3.9 | 4.2 | 4.5 | 3.7 |
| Among C-section deliveries: % without  indication | 20.6 | 21.3 | 19.1 | 17.5 | 23.7 |
| Unmet need (among women with indication: % without CS) | 6.0 | 6.0 | 6.1 | 5.4 | 5.8 |
| Among all deliveries: % with CS with indication | 13.1 | 12.4 | 14.9 | 17.4 | 10.5 |
| Among all deliveries: % with CS without indication | 3.4 | 3.4 | 3.5 | 3.7 | 3.3 |
| Among all deliveries: % with vaginal delivery with CS indication | 0.8 | 0.8 | 1.0 | 1.0 | 0.7 |
|  |  |  |  |  |  |
| *Dutch guidelines* |  |  |  |  |  |
| % of women with C-section indication | 12.5 | 11.7 | 14.7 | 17.0 | 9.2 |
| C-section rate among women with indication | 96.7 | 96.2 | 96.9 | 98.2 | 98.4 |
| C-section rate among women without indication | 5.1 | 5.1 | 4.9 | 5.3 | 5.2 |
| Among C-section deliveries: % without  indication | 27.0 | 28.4 | 22.6 | 20.7 | 34.2 |
| Unmet need (among women with indication: % without CS) | 3.3 | 3.8 | 3.1 | 1.8 | 1.6 |
| Among all deliveries: % with CS with indication | 12.1 | 11.3 | 14.3 | 16.7 | 9.1 |
| Among all deliveries: % with CS without indication | 4.5 | 4.5 | 4.2 | 4.4 | 4.7 |
| Among all deliveries: % with vaginal delivery with CS indication | 0.4 | 0.4 | 0.5 | 0.3 | 0.1 |
|  |  |  |  |  |  |
| *English guidelines* |  |  |  |  |  |
| % of women with C-section indication | 12.7 | 12.0 | 15.0 | 17.2 | 9.2 |
| C-section rate among women with indication | 96.6 | 96.1 | 96.9 | 98.2 | 98.4 |
| C-section rate among women without indication | 4.9 | 4.8 | 4.5 | 5.1 | 5.2 |
| Among C-section deliveries: % without  indication | 25.7 | 26.9 | 20.9 | 20.0 | 34.2 |
| Unmet need (among women with indication: % without CS) | 3.4 | 3.9 | 3.1 | 1.8 | 1.6 |
| Among all deliveries: % with CS with indication | 12.3 | 11.5 | 14.6 | 16.9 | 9.1 |
| Among all deliveries: % with CS without indication | 4.3 | 4.3 | 3.8 | 4.2 | 4.7 |
| Among all deliveries: % with vaginal delivery with CS indication | 0.4 | 0.5 | 0.5 | 0.3 | 0.1 |
|  |  |  |  |  |  |
| *Any of the above guidelines* |  |  |  |  |  |
| % of women with C-section indication | 15.4 | 14.6 | 17.5 | 20.3 | 12.3 |
| C-section rate among women with indication | 92.8 | 92.8 | 92.6 | 94.3 | 93.5 |
| C-section rate among women without indication | 2.6 | 2.6 | 2.6 | 2.4 | 2.7 |
| Among C-section deliveries: % without  indication | 13.3 | 14.0 | 11.8 | 9.1 | 16.8 |
| Unmet need (among women with indication: % without CS) | 7.2 | 7.2 | 7.4 | 5.7 | 6.5 |
| Among all deliveries: % with CS with indication | 14.3 | 13.6 | 16.2 | 19.2 | 11.5 |
| Among all deliveries: % with CS without indication | 2.2 | 2.2 | 2.2 | 1.9 | 2.3 |
| Among all deliveries: % with vaginal delivery with CS indication | 1.1 | 1.1 | 1.3 | 1.2 | 0.8 |
|  |  |  |  |  |  |

**Table S4:** Socioeconomic inequalities (measured in odds ratios) in C-section rate, without and with adjustment for clinical indication for C-section, previous C-section, multiple birth, presentation, gestational age, maternal age, and parity (Students compared with other socioeconomic groups)

|  | Univariate | | Adjusted for clinical indication | | Adjusted for previous C-section | | Adjusted for multiple birth, presentation, and gestational age | | Adjusted for maternal age and parity ^a^ | | Adjusted for maternal clinical indication, previous C-section, age, and parity ^a^ | | Adjusted for all ^a^ | |
| --- | --- | --- | --- | --- | --- | --- | --- | --- | --- | --- | --- | --- | --- | --- |
|  | OR [95% CI] | P value | OR [95% CI] | P Value | OR [95% CI] | P value | OR [95% CI] |  | OR [95% CI] | P Value | OR [95% CI] | P Value | OR [95% CI] | P value |
| *Occupation* |  |  |  |  |  |  |  |  |  |  |  |  |  |  |
| Housewives | 1.17 (0.99;1.38) | 0.0656 | 0.9 (0.70;1.17) | 0.43733 | 0.86 (0.72;1.02) | 0.0873 | 1.14 (0.94;1.40) | 0.1850 | 1.02 (0.86;1.21) | 0.8004 | 0.94 (0.72;1.22) | 0.6282 | 0.97 (0.70;1.35) | 0.8561 |
| Small business | 1.41 (1.17;1.70) | 0.0004 | 0.97 (0.72;1.31) | 0.85269 | 0.99 (0.81;1.21) | 0.9167 | 1.29 (1.02;1.61) | 0.0304 | 1.14 (0.94;1.39) | 0.1867 | 0.97 (0.71;1.32) | 0.8371 | 1.00 (0.68;1.48) | 0.9939 |
| Professional | 1.67 (1.36;2.04) | 0.0000 | 1.13 (0.81;1.56) | 0.47744 | 1.12 (0.90;1.40) | 0.3177 | 1.61 (1.26;2.05) | 0.0001 | 1.24 (1.00;1.54) | 0.0460 | 1.02 (0.72;1.43) | 0.9252 | 1.07 (0.69;1.65) | 0.7609 |
| Student | 1 |  | 1 |  | 1 |  | 1 |  | 1 |  | 1 |  | 1 |  |
| Overall p-value |  | 0.0000 |  | 0.37262 |  | 0.0105 |  | 0.0002 |  | 0.0413 |  | 0.9102 |  | 0.9484 |

**Figure S1: Women without previous C-section**

| **A:** C-section rate for the total population and by socioeconomic position (table), and split out by presence of clinical indication (figure) (women without previous C-section) | |
| --- | --- |
| \|  \| CS rate \| \| \| --- \| --- \| --- \| \|  \| n \| % \| \| Total population \| 1419/11605 \| 12.2 \| \| *Occupation* \|  \|  \| \| Housewife \| 779/6782 \| 11.5 \| \| Small business \| 264/2026 \| 13.0 \| \| Professional \| 174/1203 \| 14.5 \| \| Student \| 179/1364 \| 13.1 \| | Unmet need: percentage of women with C-section indication delivering vaginally  Not indicated CS: percentage of women with not medically indicated C-section  Indicated CS: percentage of women with medically indicated C-section, not medically indicated C-section |
| **B:** Percentage with indication for C-section and mode of delivery by presence of indication (women without previous C-section)   \|  \| Women with indication \| \| C-section rate among women with indication \| \| Vaginal delivery rate among women with indication \| \| C-section rate among women without indication \| \| \| --- \| --- \| --- \| --- \| --- \| --- \| --- \| --- \| --- \| \|  \| n \| % \| n \| % \| n \| % \| n \| % \| \| Total population \| 829/11605 \| 7.1 \| 803 /829 \| 96.9 \| 26 /829 \| 3.1 \| 616 /10776 \| 5.7 \| \| Housewife \| 450/6782 \| 6.6 \| 432 /450 \| 96.0 \| 18 /450 \| 4.0 \| 347 /6332 \| 5.5 \| \| Small business \| 160/2026 \| 7.9 \| 157 /160 \| 98.1 \| 3 /160 \| 1.9 \| 107 /1866 \| 5.7 \| \| Professional \| 104/1203 \| 8.6 \| 102 /104 \| 98.1 \| 2 /104 \| 1.9 \| 72 /1099 \| 6.6 \| \| Student \| 106/1364 \| 7.8 \| 104 /106 \| 98.1 \| 2 /106 \| 1.9 \| 75 /1258 \| 6.0 \| | |
| **C**: Socioeconomic differences in medical indication (women without previous C-section)   \|  \| Univariate \| \| Adjusted for maternal age and parity \| \| \| --- \| --- \| --- \| --- \| --- \| \|  \| OR [95% CI] \| P value \| OR[95% CI] \| P Value \| \| *Occupation* \|  \|  \|  \|  \| \| Housewives \| reference \|  \| reference \|  \| \| Small business \| 1.21 (1.00;1.46) \| 0.050 \| 1.17 (0.97;1.41) \| 0.107 \| \| Professional \| 1.33 (1.07;1.66) \| 0.012 \| 1.17 (0.93;1.48) \| 0.167 \| \| Student \| 1.19 (0.95;1.48) \| 0.129 \| 1.19 (0.94;1.49) \| 0.140 \| \| Overall p-value \|  \| 0.028 \|  \| 0.183 \| | |
| **D:** Distribution of C-section deliveries according to indication (women without previous C-section)   \|  \| Among CS deliveries \| \| \| \| \| \| \| \| \| \| \|  \| \|  \| \| \| --- \| --- \| --- \| --- \| --- \| --- \| --- \| --- \| --- \| --- \| --- \| --- \| --- \| --- \| --- \| --- \| \|  \| % with NO C-section indication \| \| % with only foetal distress as indication \| \| % with only prolonged labour as indication \| \| % with other indication \| \| % with multiple indications \| \| Total \| \|  \| \| \|  \| n \| % \| n \| % \| n \| % \| n \| % \| n \| % \| n \| \| % \| \| \| Total population \| 616/1419 \| 43.4 \| 245/1419 \| 17.3 \| 332/1419 \| 23.4 \| 145/1419 \| 10.2 \| 81/1419 \| 5.7 \| 1,419 \| \| 100 \| \| \| Housewife \| 347/779 \| 44.5 \| 127/779 \| 16.3 \| 174/779 \| 22.3 \| 86/779 \| 11.0 \| 45/779 \| 5.8 \| 779 \| \| 100 \| \| \| Small business \| 107/264 \| 40.5 \| 52/264 \| 19.7 \| 62/264 \| 23.5 \| 29/264 \| 11.0 \| 14/264 \| 5.3 \| 264 \| \| 100 \| \| \| Professional \| 72/174 \| 41.4 \| 36/174 \| 20.7 \| 39/174 \| 22.4 \| 19/174 \| 10.9 \| 8/174 \| 4.6 \| 174 \| \| 100 \| \| \| Student \| 75/179 \| 41.9 \| 28/179 \| 15.6 \| 54/179 \| 30.2 \| 11/179 \| 6.1 \| 11/179 \| 6.1 \| 179 \| \| 100 \| \| | |
| **E**: Socioeconomic inequalities (measured in odds ratios) in C-section rate, without and with adjustment for and clinical indication for C-section, multiple birth, presentation, gestational age, maternal age, and parity (women without previous C-section)  ****  *Note:* Adjustments for age and parity as continuous variables | |
